# Tricking enzymes in living cells: a mechanism-based strategy for design of DNA topoisomerase biosensors

**DOI:** 10.1186/s12951-021-01155-1

**Published:** 2021-12-07

**Authors:** Sai Ba, Guangpeng Gao, Tianhu Li, Hao Zhang

**Affiliations:** 1 School of Chemistry and Chemical Engineering, Northwestern Polytechnical University, Xi’an, 710072 China; 2 Division of Chemistry and Biological Chemistry, School of Physical and Mathematical Sciences, Nanyang Technological University, Singapore, 637371 Singapore

**Keywords:** DNA topoisomerases, Cancer diagnosis, Enzyme mechanisms, DNA nanotechnology, Diagnostic probes

## Abstract

Most activity-based molecular probes are designed to target enzymes that catalyze the breaking of chemical bonds and the conversion of a unimolecular substrate into bimolecular products. However, DNA topoisomerases are a class of enzymes that alter DNA topology without producing any molecular segments during catalysis, which hinders the development of practical methods for diagnosing these key biomarkers in living cells. Here, we established a new strategy for the effective sensing of the expression levels and catalytic activities of topoisomerases in cell-free systems and human cells. Using our newly designed biosensors, we tricked DNA topoisomerases within their catalytic cycles to switch on fluorescence and resume new rounds of catalysis. Considering that human topoisomerases have been widely recognized as biomarkers for multiple cancers and identified as promising targets for several anticancer drugs, we believe that these DNA-based biosensors and our design strategy would greatly benefit the future development of clinical tools for cancer diagnosis and treatment.

**Supplementary Information:**

The online version contains supplementary material available at 10.1186/s12951-021-01155-1.

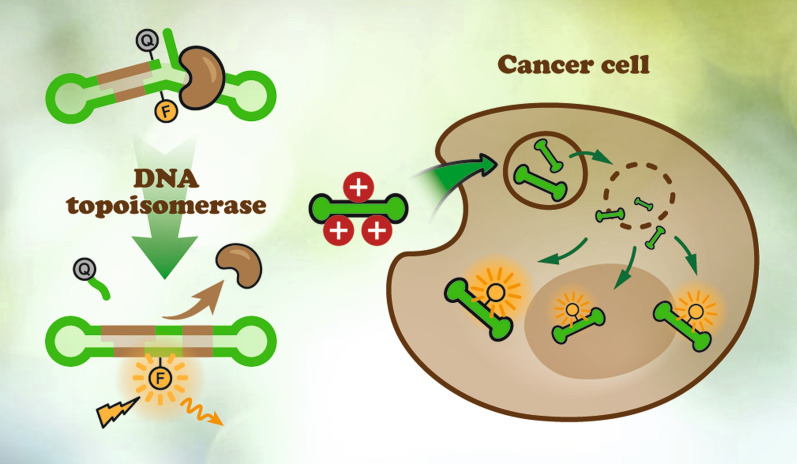

## Introduction

The catalytic action of most cellular enzymes involves the breaking of chemical bonds and the conversion of a unimolecular substrate into bimolecular products. These intrinsic properties of enzymes have been extensively utilized in the past to design fluorescent probes for sensing enzymatic behaviors in cells (Additional file 1: Figure S1a) [1–4]. For example, a peptide derivative with a caspase cleavage site and a fluorophore-quencher pair was recently synthesized [3]. As a result of its exclusive response to the catalytic action of caspase (Additional file 1: Figure S2), this peptide derivative was reported as a biosensor to quantify caspases in biological samples [3]. Another example is an iminocoumarin-benzothiazole-based structure with a covalently linked phosphate group [4]. Once the cellular alkaline phosphatase breaks the phosphate ester bond within the molecule (Additional file 1: Figure S3), quenched fluorescence is restored in living cells [4]. In addition to the aforementioned examples, a wide variety of enzymes have been detected using molecular probes designed on the basis of unimolecular to bimolecular conversion processes. [5–8].

In contrast to enzymes that catalyze the breaking of chemical bonds [1–8], topoisomerases regulate torsional stress in DNA molecules without releasing any molecular segments [9, 10], which hinders the design of fluorescent probes for these enzymes using conventional strategies (Additional file 1: Figure S1b). Thus, although detection of some topoisomerases in extracellular environment were attempted in the recent years [11–16], most of them relied intensively on the usage of inorganic materials [14, 15] (*e.g.* magnetic beads, glass) and exogenous enzymes [14–16] (*e.g.* DNA ligase, DNA polymerase, polynucleotide kinase), as well as the application of rolling circle amplification [14–16]. It was the artificially introduced substances and sophisticated in vitro techniques that substantially limited the use of these traditional methods in living cells.

Here, we report a novel strategy for efficiently diagnosing cellular DNA topoisomerases, enzymes whose natural substrates and products have identical molecular configurations. In our strategy we tricked DNA topoisomerases on a unimolecular pseudo-substrate oligonucleotide to switch on fluorescence and resume new rounds of catalysis at the same time. Additionally, replacement of some phosphodiester linkages with internal phosphorothioates significantly improved the intracellular stability of these DNA-based biosensors, which ensures their ability to function in human cells. From a clinical point of view, human topoisomerase I is overexpressed in a variety of human cancer cells and has been identified as a key biomarker for cancer diagnosis, prognosis, and monitoring therapeutic responses [17–23]. Moreover, it serves as a cellular target for several FDA-approved anticancer drugs [24, 25]. In this study, we demonstrate that our DNA-based biosensors can effectively sense differences in topo I catalytic activity among human cancer cells, normal cells, topo I gene-knockdown cells, and chemotherapy agent-treated cells. Considering that topo I is a validated biomarker for a wide range of cancers and there are no molecular tools available thus far for diagnosing topo I expression levels in living cells, we believe that our newly designed probes could have great potential for developing clinical tools for determining cancer aggressiveness [21], monitoring cancer treatment [22, 23], and evaluating an individual's predisposition for cancer. [21].

## Results and discussion

### Strategy for designing mechanism-based biosensors for dna topoisomerases

**Fig. 1:**
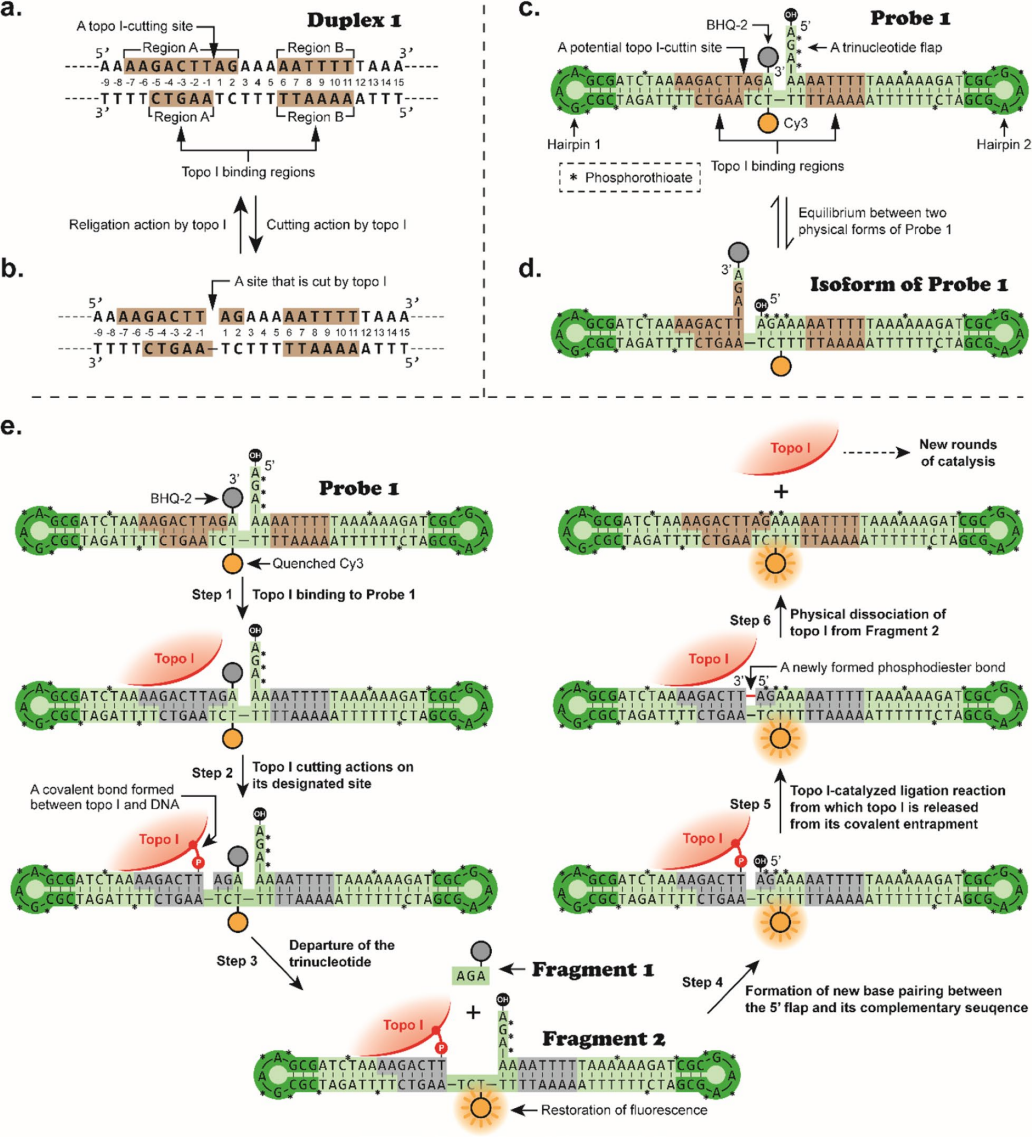
Schematic illustrations of topoisomerase I-catalyzed reactions on duplex DNA. **a** An example of a well-studied topo I-binding DNA sequence (Duplex 1) [29]. **b** The catalytic intermediate of topo I as reported by the Westergaard group [29]. In the reaction, topo I generates a nick site on Duplex 1 and reseals the nick site unceasingly. **c** A structural illustration of one of our newly designed DNA-based biosensors (Probe 1). **d** An illustration of the Probe 1 isoform that is in dynamic equilibrium with Probe 1. This equilibrium will shift to favor the formation of Probe 1 when Probe 1 is consumed by the action of topo I. **e** The catalytic mechanism of human topo I on Probe 1. Steps 1–3: Topo I is misled to act on the oligonucleotide, trigger the release of Fragment 1, and consequently switch on the quenched fluorescence. Steps 4–6: Topo I is tricked into accepting the flapped trinucleotide as part of its original substrate for proceeding with the subsequent re-ligation process. This action allows topo I to resume its original structure for new rounds of catalysis

To demonstrate the viability of our new strategy, proof-of-concept studies were performed using human topoisomerase I (topo I) as the target. Topoisomerases change DNA supercoiling by initially binding to any sequence of double-stranded DNA [26–28]. Westergaard group reported in 1985, however, human topoisomerase I does not act on all DNA sequences indiscriminately but preferentially binds to particular tracts (*e.g.*, Duplex 1 shown in Fig. 1a and b) in the macronuclear DNA of the eukaryote, Tetrahymena thermophila [29]. Since then, these particular DNA sequences have been extensively studied [30, 31], including the incorporation of such sequences into circular plasmids to facilitate DNA relaxation (Additional file 1: Figure S4) [32, 33]. Taking advantage of the aforementioned discoveries [29–31], we designed a DNA-based biosensor in our study (Probe 1 in Fig. 1c), which possesses the following characteristics:

#### Functional components incorporated in a unimolecular substrate-based oligonucleotide

In comparison with Duplex 1, Probe 1 is a derived version of the topo I substrate that possesses five additional components in its structure (Fig. 1c): (i) a fluorophore (Cy3) covalently modified near the topo I binding site, (ii) a fluorescence quencher (BHQ-2) covalently modified at the 3' terminus, (iii) a broken end with a 5'-flapped trinucleotide near the topo I cutting site, (iv) two stable hairpin loops, and (v) phosphorothioate modifications in phosphate backbones. To ensure that the modified oligonucleotide is still accessible to topo I, all modifications are incorporated at the sites where topo I is not in physical contact. Most importantly, our DNA-based biosensor is a self-assembled unimolecular structure that can, in principle, enhance its stability in living cells.

#### Fluorescence quenching prior to the action of topo I

Within the self-folding structure of Probe 1, a pair of fluorophore and fluorescence quencher merge in close proximity, which ensures that the adjacent BHQ-2 effectively quenches the fluorescence signals of Cy3 on the opposite strand.

#### Restoration of fluorescence induced by the catalytic action of topo I

Once topo I cleaves the phosphodiester bond at its specific site, this enzyme is expected to covalently link to the 3' end of DNA and hold the 5' end of DNA through hydrogen bonding as in its natural catalytic cycle [31]. As a result, a short BHQ-2-linked trinucleotide (Fragment 1 in Fig. 1e) is anticipated to depart from the main body of the probe, which will lead to the restoration of the Cy3 fluorescence signal. Thus, topo I is tricked into accepting a specially designed oligonucleotide as its substrate and initializing the dissociation of fluorophore-quencher pairs.

#### Recovery of topo I from the enzyme–substrate complex for new rounds of catalysis

A flapped trinucleotide was designed at the 5' end of Probe 1 to fill the gap generated by the action of topo I. Once the flap structure fills the gap through Watson–Crick base pairing, topo I is predicted to ligate the free 5' end with the covalently linked 3' end of DNA. In this process, topo I is tricked for the second time to take the 5’-flapped trinucleotide as the departed fragment and to liberate itself from the enzyme–substrate complex for new rounds of catalysis.

#### Enhancement of intracellular stability with phosphorothioate linkages

Phosphorothioate modifications were introduced at certain oligonucleotide positions (denoted with stars in Fig. 1c) to enhance its nuclease hydrolysis resistance. [34].

Using our newly designed mechanism-based biosensors, we tricked DNA topoisomerases twice during catalysis (Fig. 1). First, the enzyme is misled to act on a specifically designed oligonucleotide and consequently switch on the quenched fluorescence from a DNA-based probe. Then, topo I is tricked into accepting an oligonucleotide moiety disguised as part of its original substrate to proceed with the subsequent elimination process. This action allows topo I to effectively detach from the covalent conjugate and resume its original structure for new rounds of catalysis. Furthermore, it should be noted that Probe 1 is always in equilibrium with its isoform (Fig. 1d). This equilibrium shifts to favor the formation of Probe 1 when it is consumed by the action of topo I in living cells. In addition to Probe 1, we designed other probes (Probe 2–7) for comparison purposes. Detailed nucleotide sequences, synthetic procedures, and characterizations for all the probes are provided in the Supporting Information (Additional file 1: Table S1; Figures S5–S8). As the fluorophore on Probe 1 is located close to the quencher but far away from the topo I’s binding site, Probe 1 exhibited the highest reactivity toward human topo I among all the probes (Additional file 1: Figure S9) and was employed in our subsequent studies. In principle, this mechanism-based strategy, with certain adaptations, should be suitable for designing molecular probes for DNA topoisomerases and other cellular enzymes that cause no changes in atomic connectivity during catalysis.

### Validation of our newly designed biosensors in cell-free systems

**Fig. 2:**
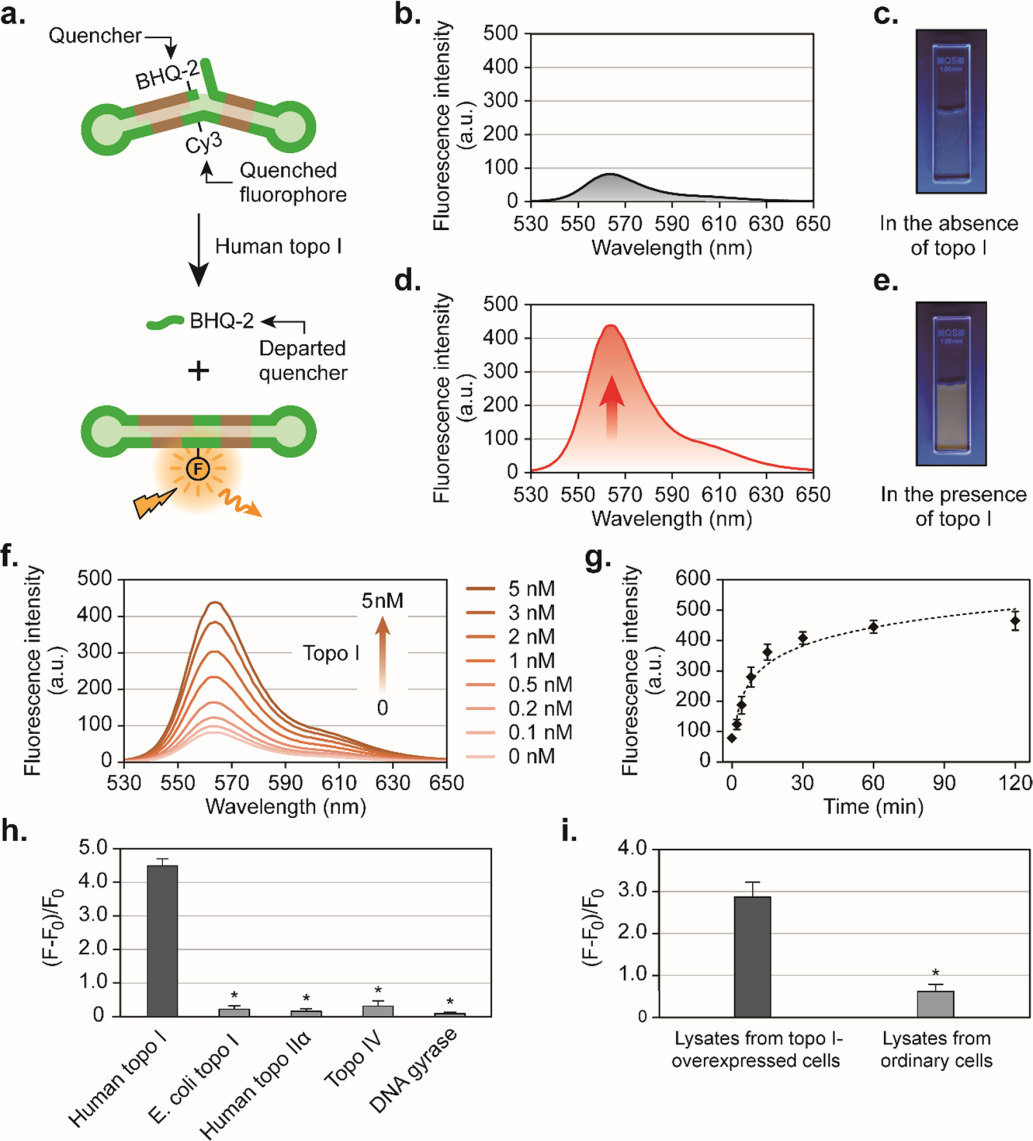
Examination of the sensitivities of the newly designed biosensors to human topo I in cell-free systems. **a** Illustration of topo I-activated restoration of fluorescence from Probe 1. **b** The fluorescence spectrum of Probe 1 in the absence of topo I. **c** Colorimetric study of Probe 1 under UV irradiation in the absence of topo I. **d** The fluorescence spectrum of Probe 1 in the presence of topo I. **e** Colorimetric study of Probe 1 under UV irradiation in the presence of topo I. **f** The fluorescence spectra of Probe 1 in the presence of different concentrations of topo I. **g** Correlation of fluorescence intensities with the incubation times of Probe 1 and topo I. **h** Examination of the specificity of Probe 1 for other non-target topoisomerases. **i** Examination of the performance of Probe 1 in protein lysates of topo I-overexpressed cells and ordinary cells. Unless otherwise stated, all reactions were performed with 50 nM Probe 1 and 5 nM human topo I at 37 °C for 1 h in aluminum-foil-protected tubes. Detailed experimental procedures are described in Supporting Information. Data shown in g, h, and i are expressed as the mean ± standard deviation (*P < 0.005; n = 5)

To determine whether the DNA-based biosensors are accessible and photoswitchable by topo I's catalytic action as originally designed, fluorescence spectroscopic examinations were conducted (Fig. 2). As seen in Fig. 2b and d, the fluorescence intensity was greatly induced by topo I treatment. In addition to these spectroscopic examinations, colorimetric studies also revealed that Probe 1, which was treated with topo I, yielded an orange color under UV irradiation (Fig. 2c and e). In separate studies, enhancement of the fluorescence intensity of Probe 1 was observed with increasing molar concentrations of topo I (Fig. 2f) and reaction time (Fig. 2g). All the above-mentioned results indicated that our newly designed Probe 1 could be used to quantify the concentration of topo I in cell-free systems. In order for the assay to be quantitative, mean fluorescence intensity values at each concentration were obtained (Table 1), from which the limit of detection (LOD) of Probe 1 was determined to be 57 pM. In principle, low-molecular-weight DNA segments should be generated upon topo I catalysis. To verify that the molecular structure of Probe 1 was indeed fragmented, a gel mobility shift assay was performed. As shown in Additional file 1: Figure S10, with an increasing concentration of human topo I, most of the oligonucleotides were cleaved while lower molecular weight fragments were generated.

**Table 1:** Relationship between fluorescence intensity measured at 565 nm and concentration of the analyte human topo I

Topo I concentration/nM	Mean fluorescence intensity	Standard deviation
0	81.6	3.9
0.1	97.8	4.2
0.2	120.8	11.1
0.5	162.4	15.1
1	232.1	22.6
2	303.0	18.0
3	380.9	22.8
5	436.9	26.5

Even though Probes 2 and 3 closely resemble Probe 1 in their structures, none of these probes are designed to be the substrate of human topo I (Additional file 1: Figure S11). Probe 2 is different from Probe 1 because it lacks a flap structure at its 5' end. As shown in the fluorescence spectra shown in Additional file 1: Figure S11d, this probe could not generate fluorescence signals in the presence of topo I. This observation corroborates that the presence of 5' flap structures in Probe 1 is indispensable for recovering trapped topo I in its catalysis (Steps 4–6 in Fig. 1e). Additionally, Probe 3 was examined in our studies, whose structure was the same as Probe 1 except that a phosphate group was modified at its 5' end. As seen in Additional file 1: Figure S11f, a negligible amount of fluorescence signal emerged upon incubation of Probe 3 with topo I, which is consistent with the suggestion that the phosphate at the 5' end of Probe 3 prevents the re-ligation reaction (Step 5 in Fig. 1e) of topo I from occurring. To investigate whether our newly designed probes are human topo I-specific, we examined the reactions of Probe 1 with E. coli topo I, human topo IIα, topo IV, DNA gyrase, and human topo I in parallel, which are all DNA supercoil-altering enzymes [26]. As shown in Fig. 2h, all of these enzymes caused trivial enhancement of fluorescence signals except for human topo I, which is an indication that Probe 1 is highly specific to human topo I. All of the observations mentioned above are consistent with the notion that Probe 1 effectively induced human topo I in buffer solutions to switch on the quenched fluorescence.

### Evaluation of the performance of DNA-based biosensors in cell lysates

To determine how diverse types of intracellular proteins derived from human cells affect the DNA-based probes, protein lysates of ordinary HEK293T cells and topo I-upregulated HEK293T cells were treated separately with Probe 1. As shown in Fig. 2i, the protein lysates from ordinary cells led to the generation of low fluorescence signals, which corroborates that the proteins derived from these cells are incapable of causing effective chemical changes in Probe 1. In contrast to ordinary HEK293T cells, the protein lysates from topo I-upregulated HEK293T cells drove Probe 1 to produce significantly high fluorescence signals. In addition, ELISA assays were performed to quantify the levels of topo I in lysates of the topo I-upregulated HEK293T cells as well as the ordinary HEK293T cells (Additional file 1: Figure S12). These results suggest that our newly designed mechanism-based biosensors can be applied to purified analytes and human cell lysates containing overexpressed proteins.

### Examination of the responsiveness and specificity of DNA-based biosensors in living cells

**Fig. 3:**
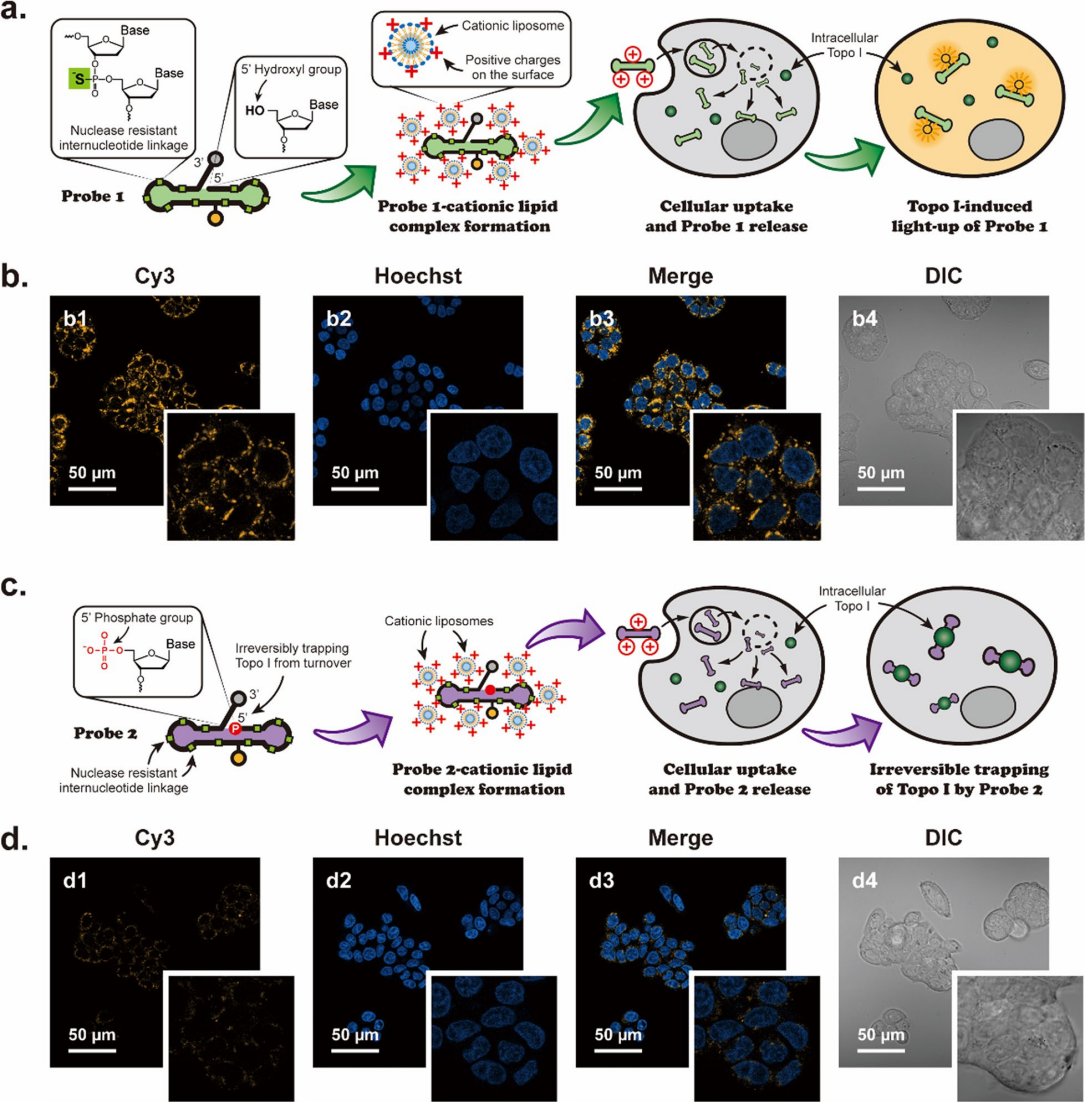
Examination of the responsiveness of the DNA-based biosensors in human cancer cells. **a** Schematic illustration of Probe 1 for sensing of human topo I in living cells. **b** Confocal laser scanning microscopy images of Probe 1-treated cancer cells. The high fluorescence intensity in Cy3 channel (b1) indicates the successful restoration of fluorescence from Probe 1 by catalytic actions of intracellular topo I. **c** Schematic illustration of actions of Probe 2 in living cells. **d** Confocal laser scanning microscopy images of Probe 2-treated cancer cells. The low fluorescence intensity in the Cy3 channel (d1) indicates that without the critical 5' hydroxyl group, Probe 2 did not generate fluorescence even in the presence of intracellular topo I. Cells were incubated with 200 nM probes at 37 °C for 36 h prior to confocal examination. Nuclei were stained with Hoechst 33342

Upon validating their designed roles in buffer solutions and cell lysates, we subsequently examined whether these DNA-based biosensors could trick intracellular topoisomerases inside human living cells (Fig. 3). The human colon cancer cell line HT-29 was chosen for our subsequent studies, which is known to overexpress topo I in cellular environments [35]. Probe 1 was introduced into living cells using liposomes as carriers [36] (Fig. 3a) followed by confocal laser scanning microscopy. The results shown in Fig. 3b verified that intracellular topo I could effectively switch on the quenched fluorescence from Probe 1. To investigate whether the DNA-based biosensors and the cationic liposome nanocarriers could induce cytotoxic effects in human cells, MTT assays were performed to evaluate the viability of HT-29 cells at 36 h after exposure, from which no significant cytotoxicity was observed at concentrations up to 200 nM of Probe 1 (Additional file 1: Figure S13). These results demonstrate that our newly designed DNA-based probes are compatible with cytoplasmic environments, especially in the presence of different types of metal ions, high concentrations of glutathione, and varied pH values. In other words, the cytoplasmic compatibility of these probes allows them to function as efficient substrates for intracellular topo I inside human cells.

In addition to Probe 1, HT-29 cells were incubated with Probe 2 during our investigations for comparison purposes (Fig. 3c). The structural difference between Probe 1 and Probe 2 is that there is a phosphate group modified on the 5’ end of Probe 2 which is not present on Probe 1. Due to the structural difference, the new phosphodiester bond could not form at the nick site of the enzyme-Probe 2 complex, which hinders the release of topo I from the complex for new rounds of catalysis. The negligible fluorescence signal in Fig. 3d suggested that neither topo I nor other cellular enzymes could chemically dissociate the fluorophore-quencher pair in Probe 2. Since these Probe 1 and Probe 2 share similar structures except for a 5' phosphate group but showed different efficacy profiles in living cells, we conclude that the fluorescence observed from Probe 1-treated cells was solely generated by catalytic activity of the overexpressed intracellular topo I.

### Determination of cellular DNA topoisomerase levels among various types of human cells using our newly designed biosensors

**Fig. 4:**
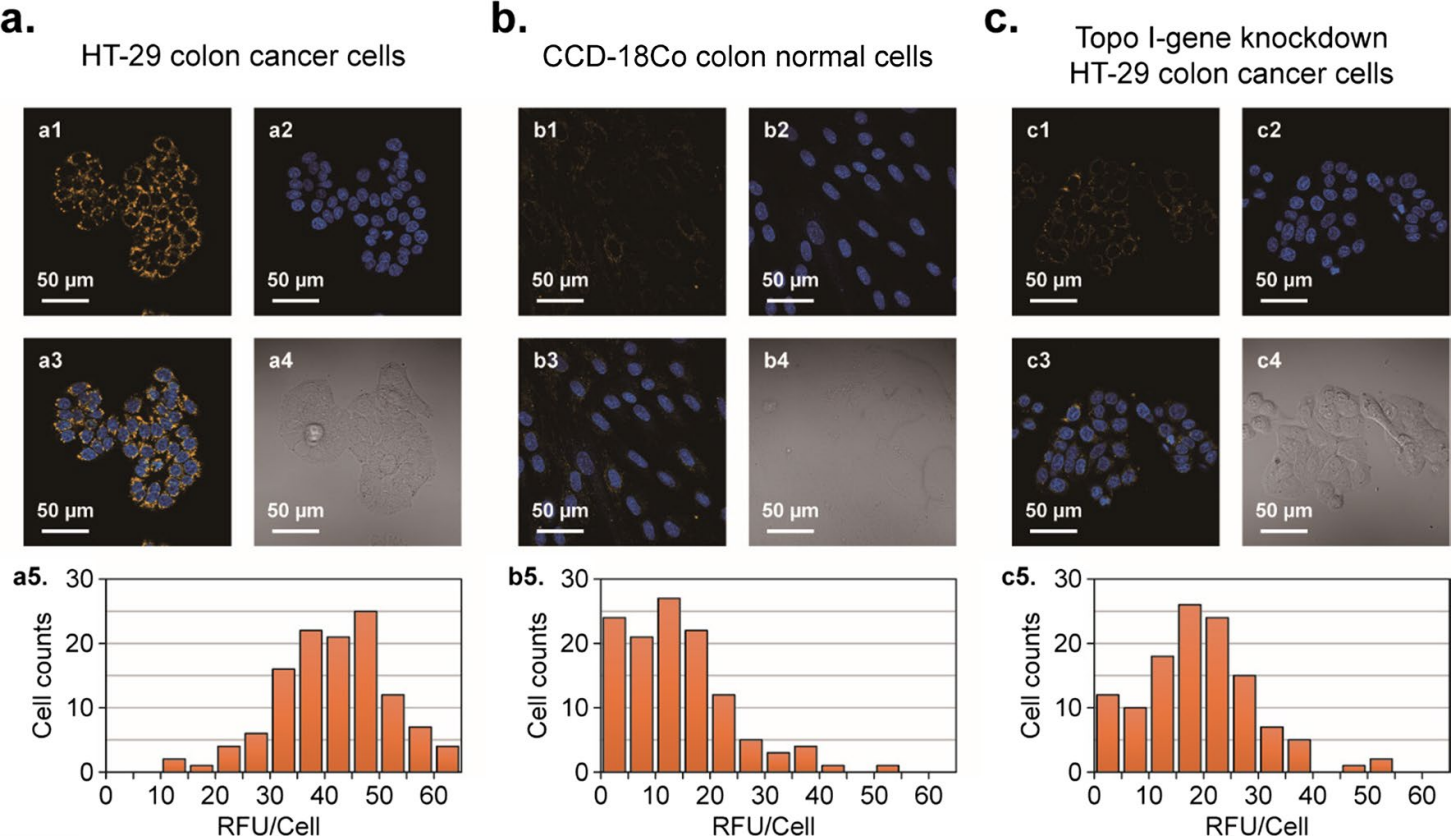
Determination and comparison of cellular DNA topoisomerase levels among HT-29 colon cancer cells (**a**), CCD-18Co colon normal cells (**b**), and topo I gene-knockdown HT-29 cells (**c**) using our newly designed biosensors. Cells were incubated with 200 nM probes at 37 °C for 36 h prior to confocal examination. Nuclei were stained with Hoechst 33,342. Relative fluorescence intensity per cell (RFU/Cell) was quantified from microscopic images with the areas manually selected (n = 120 cells) using ImageJ. The observed high fluorescence intensity in a1 is affiliated with actions of highly abundant topo I in colon cancer cells HT-29, while the observed low fluorescence intensities in b1 and c1 are affiliated with insufficient amounts of topo I in colon normal cells and topo I gene-knockdown cells to activate Probe 1

As a cancer diagnostic, prognostic, and predictive biomarker, human topoisomerase I is expressed differently between cancerous and healthy cells [35, 37]. To determine whether our newly designed biosensors could serve as effective tools for diagnosing different topo I expression levels between normal and cancer cells, we examined CCD-18Co (colon normal cells) and HT-29 (colon cancer cells) in parallel during our investigations (Fig. 4). As seen in the confocal laser scanning microscopy images (Fig. 4a and b), colon normal cells displayed lower fluorescence intensity than HT-29 colon cancer cells. These differences suggest that, unlike colon cancer cells, the amount of topo I in their healthy counterparts was insufficient to generate high fluorescence signals from the probes.

In addition to the aforementioned HT-29 cells, topo I gene-knockdown HT-29 cells were constructed in our lab following previously reported protocols [38]. Validation of the knockout of topo I in HT-cells were performed via ELISA assays (Additional file 1: Figure S14). To verify whether our probes could be used for determining topo I expression levels in gene knockout cells, these two types of cells, native and topo I gene-silenced HT-29, were incubated with Probe 1 in parallel. As shown in Fig. 4c, the fluorescence intensity of gene-knockdown cells was considerably lower than that of native HT-29 cells, indicating insufficient intracellular topo I in the gene-knockdown cells to activate the probes.

Based on the comparison studies between these colon cells, we suggest that our DNA-based probes, as well as our design strategies, could benefit the future development of diagnostic tools for monitoring cellular DNA topoisomerase levels and other biomarkers among various living cells for determining cancer aggressiveness, and for evaluating an individual's predisposition to cancers.

### Examination of the effects of FDA-approved chemotherapeutic agents on topoisomerase enzymatic activity using our newly designed biosensors

**Fig. 5:**
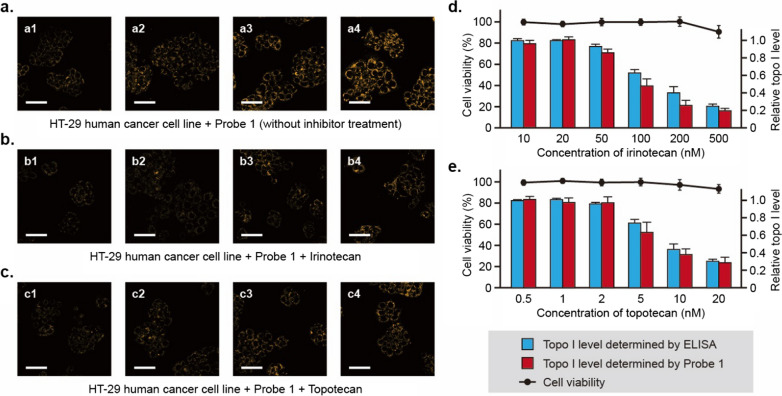
Examination of the effects of organic inhibitors on topo I’s enzymatic activity in living cells using our newly developed biosensor Probe 1. **a**–**c** HT-29 human cancer cells were pre-mixed with PBS (**a**), 500 nM of irinotecan (**b**) or 20 nM of topotecan (**c**) prior to incubation with 200 nM of Probe 1 at 37 °C for 4.5 h (a1, b1, c1), 9 h (a2, b2, c2), 18 h (a3, b3, c3) and 36 h (a4, b4, c4) respectively. Confocal examinations were carried out according to the procedures described in Supporting Information. All scale bars represent 50 μm in this figure. **d**, **e** Effects of topo I inhibitors on cell viability and determined levels of cellular topo I. HT-29 cells were pre-mixed with 10, 20, 50, 100, 200 and 500 nM of irinotecan (**d**) or 0.5, 1, 2, 5, 10, 20 nM of topotecan (**e**) followed by incubation with 200 nM of Probe 1 at 37 °C for 36 h. Cell viability assays were carried out through using MTT cell proliferation assay kits, results of which were plotted as the mean ± SD (n = 3, black dots). Determination of topo I levels using Probe 1 was performed according to the procedures described in Supporting Information and shown as the mean ± SD (n = 3, red bars). Determination of topo I levels using ELISA kits was performed according to the manufacturer’s protocol and shown as the mean ± SD (n = 3, blue bars)

As a treatment response biomarker, the expression of DNA topoisomerase I often fluctuates in cancer cells before and after anticancer drug treatment [17–23]. Irinotecan, an FDA-approved anticancer drug and a potent inhibitor of human topo I [39], was subsequently used in our study to treat human HT-29 cancer cells (Fig. 5). As shown in Fig. 5a, HT-29 cells without irinotecan treatment displayed significantly higher fluorescence intensity. However, after irinotecan treatment, the resultant cancer cells showed only a trivial amount of fluorescence (Fig. 5b). These observations are consistent with the proposition that in the presence of the topo I inhibitor, the catalytic activity of intracellular topo I was drastically suppressed, which incapacitates catalysis of topo I on the probes inside living cells. It can be deduced from these observations that HT-29 colon cancer cells are susceptible to irinotecan treatment because of the greatly reduced cellular topo I activity. Besides irinotecan, another FDA-approved anticancer drug and inhibitor of topo I topotecan [24, 25] was examined also during our investigation. Similar to the results obtained from irinotecan studies, cancer cells before and after topotecan treatment were discernable using Probe 1 as a diagnostic tool (Fig. 5c).

In addition, intracellular levels of topo I were measured by a commercially available ELISA kit in our study as well, results of which (blue bars in Fig. 5d and e) were compared with those determined by Probe 1 (red bars in Fig. 5d and e). Taken together, these results revealed that our newly developed DNA-based biosensors could be used for effectively diagnosing intracellular topo I as well as a standard ELISA. Furthermore, MTT assays were performed to investigate the viability of HT-29 cells. As shown in Fig. 5d and e, even the topo I level decreased significantly with increasing concentration of topo I inhibitors, the resultant cell viabilities still remained above 90% (black dots), which suggested that the observed difference in fluorescence between topo I inhibitor-treated and untreated cells is mainly due to the change in topo I levels rather than in cell viabilities. Based on our examination of the effects of chemotherapy agents on topo I activity, we propose that our DNA-based biosensors could be developed as useful clinical tools for monitoring anticancer treatment responses using a small number of human cells.

## Conclusion

In this study, we established a new strategy for the effective sensing of DNA topoisomerases and monitoring their catalytic activities in living cells. In contrast to most conventional molecular probe designs, our mechanism-based biosensors involve an 89-mer oligonucleotide structure to mimic the natural substrate of DNA topoisomerases. Taking advantage of the unique mechanism of topoisomerases, we tricked the enzymes within their catalytic cycles twice to (i) switch on fluorescence from the oligonucleotides and (ii) resume the original enzymatic process for new rounds of catalysis. Thus, even though the natural substrates and products of DNA topoisomerases share identical molecular configurations, these cancer biomarkers can be effectively diagnosed in cell-free systems as well as in living cells. Considering that human topoisomerases are recognized to be overexpressed in multiple cancers (*e.g.* colorectal cancer, ovarian cancer, leukemia) and recently identified as promising targets for several anticancer drugs, we believe that our newly designed biosensors could have great potential for development into clinical tools for determining aggressiveness of certain types of cancers, monitoring cancer treatment, and evaluating an individual's predisposition to developing cancers in the future.

## Experimental section

### Preparations of human topo I switch-on fluorescence probes

For synthesis of Probe 1, a solution containing 10 μM of Oligonucleotide 1, 10 mM Tris–HCl (pH 7.5) and 50 mM NaCl was incubated at 95 °C for 5 min and was further allowed to cool to 25 °C over a time period of 2 h to let the oligonucleotide molecules to form self-annealing structures. The same procedures were followed for preparation of self-annealing Oligonucleotide 2 as well. The two self-annealing structures were mixed in a molar ratio of 1: 1 in the presence of 50 mM Tris–HCl (pH 7.5), 5 mM NaCl, 10 mM MgCl_2_, 1 mM ATP, 10 mM DTT and further incubated at room temperature for 1 h. To the resultant mixture, 80 units of T4 DNA ligase were added, which was further kept at 16 °C for 2 h to allow the formation new phosphodiester bonds between Oligonucleotide 1 and Oligonucleotide 2 (Additional file 1: Figure S5). QIAquick nucleotide removal kit (QIAGEN) was then used for purification of newly formed Probe 1 from its reaction mixtures. For synthesis of Probe 2 and Probe 3, Probe 2 was prepared through following the same procedures as those for preparing Probe 1 except that Oligonucleotides 2 was replaced with Oligonucleotide 3. In addition, the procedures for preparation of Probe 3 were identical to those for synthesizing Probe 1 as well except that Oligonucleotide 4 was used to replace Oligonucleotide 2.

### Fluorescence spectroscopic analysis

For conducting the studies as shown in Fig. 2b and d , a solution containing 50 nM of Probe 1, 10 mM Tris–HCl (pH 7.5), 1 mM EDTA, 100 mM NaCl, 0.1% BSA, 0.1 mM spermidine, and 5% glycerol was kept at 37 °C for 1 h in aluminum-foil-protected microcentrifuge tubes in the absence (Fig. 2B) or presence of (Fig. 2D) 5 nM of human topo I. Fluorescence spectroscopic examinations were carried out at 37 °C in an ultra-micro quartz cuvette (3 × 3 mm light path) using an RF-5301PC fluorescence spectrometer (Shimadzu). Fluorescence emission spectra were measured at an angle of 90° to the 515 nm excitation laser and recorded for every nanometer from 530 to 650 nm. Both excitation and emission slits were set at 5 nm. For conducting the studies as shown in Fig. 2f, the same procedures as those for conducting the studies shown in Fig. 2d were followed except that different concentrations (0, 0.1, 0.2, 0.5, 1, 2, 3, or 5 nM) of topo I were used. For conducting the studies as shown in Fig. 2g, the same procedures as those for conducting the studies shown in Fig. 2d were followed except that reaction times were set as 2, 4, 8, 15, 30, 60, 120 min respectively. For conducting the studies as shown in Fig. 2h, the same procedures as those for conducting the studies shown in Fig. 2d were followed except that different enzymes (*E. coli* topoisomerase I, human topoisomerase IIα, topoisomerase IV, and DNA gyrase) were used. For conducting the studies as shown in Fig. 2i, the same procedures as those for conducting the studies shown in Fig. 2d were followed except that human topo I was replaced with protein lysates (10 µg) of RC21565-transfected human HEK293T cells and ordinary HEK293T cells respectively. As for the statistical analysis reported in Fig. 2h and i, F0 and F represent intensities of the fluorescence at the wavelength of 565 nm before and after enzymatic reactions, respectively. The value of (F − F0) / F0 indicates the change in fluorescence intensity relative (F − F0) to the baseline (F0). Data shown are expressed as the mean ± standard deviation (n = 5). Intracellular levels of human topo I in RC21565-transfected and ordinary HEK293T cells were determined using Enzyme-linked Immunosorbent Assay (ELISA) kits for topo I through following manufacturer’s protocol.

### Determination of limit of detection (LOD) of probe 1

In order for the assay to be quantitative, mean fluorescence intensity values at each concentration along with the standard deviation are collected from three replicates. A standard fitting curve was accordingly constructed as described below based on the data presented in the following table (Table 1):

$$Fitting \, equation: \, y \, = \, A \, {-} \, B \, * \, ln \, \left( {x \, + \, C} \right), \, in \, which \, A \, = \, 262.9, \, B \, = \, - 88.2, \, C \, = \, 0$$

Based on the previously reported method for determination of limit of detection (LoD) [40], the LoD for Probe 1 was calculated as follows:

*Mean blank = 0.051 pM*

*SD blank = 0.002 nM*

*SD low concentration sample = 0.002 nM*

*LoB = Mean blank + 1.645(SD blank) = 0.054 nM*

*LoD = LoB + 1.645(SD low concentration sample) = 0.057 nM = 57 pM*

### Colorimetric imaging under ultraviolet illumination

A solution containing 10 mM Tris–HCl (pH 7.5), 1 mM EDTA, 100 mM NaCl, 0.1% BSA, 0.1 mM spermidine, and 5% glycerol in the presence of 5 nM of topo I (Fig. 2e) and 0 nM of topo I (Fig. 2c) was incubated at 37 °C for 1 h. The reactions were stopped by heating the samples at 65 °C for 15 min. Upon their condensation under vacuum, the resultant samples were transferred to quartz cuvettes (Hellma 100–1-20 QS). Photographs of reaction mixtures in quartz cuvettes were obtained by using a Sony A7II digital camera under ultraviolet illumination (302 nm) in a dark room.

### Cell culture

HT-29 human colon cancer cells and siRNA-treated HT-29 cells were cultivated in McCoy's 5A (modified) medium supplemented with 10% (v/v) fetal bovine serum. CCD-18Co non-tumorigenic human colon cells were cultured in Eagle's Minimum Essential Medium (EMEM) supplemented with 10% (v/v) fetal bovine serum. All the cells were cultured at 37 °C in a Forma 371 Steri Cycle incubator (Thermo Fisher Scientific) under a humidified atmosphere of 5% carbon dioxide. Replacement of growth medium was carried out every two to three days during the cultivation periods. After reaching 80–90% confluency, the cells were trypsinized with 0.25% trypsin–EDTA and subcultured at a ratio of 1: 4.

### Preparation of topo I gene-knockdown HT-29 cells

Topo I gene-knockdown HT-29 cells were obtained by transfection of chemically synthesized small interfering RNA (ON-TARGETplus Human TOP1 siRNA) following the manufacturing protocols. Briefly, stock solutions of Lipofectamine RNAiMAX and siRNA were mixed in separate microcentrifuge tubes using serum-free media as solvents at room temperature using a volume ratio of 1: 1. The resultant RNA/lipid mixture was further incubated at room temperature for 5 min. The resultant mixture was then incubated with HT-29 cells at room temperature for 48 h.

### Confocal laser scanning microscopic studies

Cultured cells were seeded in 35-mm imaging dishes (Ibidi µ-Dishes) and further allowed to grow at 37 °C in the presence of 5% carbon dioxide to yield a final cell density of 500 cells/mm^2^. Probe 1 (200 nM) were pre-mixed with Lipofectamine LTX and PLUS Reagent according to the manufacturing protocol followed by incubating them with cultured HT-29 cells at 37 °C for 36 h. These samples were then gently removed and the remaining cells were washed twice with phosphate-buffered saline (pH 7.4). When it was necessary, two drops of NucBlue Live ReadyProbes reagent were incubated with the acquired cells for extra 10 min for the nuclear staining purpose. Confocal laser scanning microscopy was carried out on an LSM 800 confocal microscope (Zeiss) coupled with 40 × or 100 × oil immersion objectives. Relative fluorescence intensity per cell was quantified by the mean grey value of microscopy images with the areas manually selected (n = 120 cells) using an open-source software ImageJ. For conducting the studies reported in Fig. 3c, the same procedures as those for conducting the studies as shown in Fig. 3b were followed except that Probe 1 was replaced with Probe 2. For conducting the studies reported in Fig. 3e, the same procedures as those for conducting the studies as shown in Fig. 3b were followed except that HT-29 cells were replaced with CCD-18Co cells. For conducting the studies reported in Fig. 3f, the same procedures as those for conducting the studies as shown in Fig. 3b were followed except that HT-29 cells were replaced with topo I gene knockdown HT-29 cells.

### Cell viability assay

Colorimetric cell viability assays were performed by Vybrant MTT cell proliferation assay kits following the manufacturer’s protocols. Briefly, cells were seeded on 96-well plates at a density of ~ 5,000 cells/well for 48 h in complete culture media. MTT was then added to each well to reach a final concentration of 0.5 mg/ml, which were subsequently incubated at 37 °C for 4 h. After that, most of the culture media were removed from the wells followed by adding DMSO to dissolve formazan crystals. Optical densities of the obtained solutions were recorded using an Infinite M200 monochromator-based microplate reader (Tecan) at wavelength of 540 nm, values of which are presented as means of three independent replicates ± standard deviation (SD).

### Enzyme-Linked immunosorbent (ELISA) assay

Intracellular levels of topo I in human cells were quantified using ELISA kits for topo I (Cloud-Clone, TX) following the manufacturer’s protocol. In brief, cells were detached by treatment with trypsin and the cell debris were removed by centrifugation at 10,000 rpm for 10 min. The obtained cell lysates were added into a 96-well strip plate pre-coated with topo I antibodies for ELISA analysis. Measurements were conducted using an Infinite M200 monochromator-based microplate reader (Tecan) at wavelength of 450 nm, results of which are presented as relative values of three independent replicates ± standard deviation. Relative expression levels were determined by comparing the levels of topo I in gene down- and up-regulated cells.

## Supplementary Information

**Additional file 1: Figure S1**. Comparison between enzymatic reactions catalyzed by most cellular enzymes and DNA topoisomerases. (a) Schematic illustration of an enzyme-catalyzed reaction starting with fluorophore-quencher-containing substrates. In this process, fluorescence signals of a quenched fluorophore recommence owing to generation of bimolecular entities. (b) Schematic illustration of a human topoisomerase-catalyzed reaction. In the catalytic reaction, DNA topoisomerases causes conformational differences between its substrate (supercoiled form DNA) and product (relaxed form DNA) while molecular configurations of them remain unchanged. **Figure S2**. Caspase-catalyzed conversion of a unimolecular peptide derivative to biomolecular segments as reported by Kool group.[3] Because cellular caspase-catalyzed covalent bond breakage resulted in separation of tetrapyrene from dabcyl, quenched fluorescence signals of tetrapyrene recommenced in living cells. **Figure S3**. Alkaline phosphatase-catalyzed conversion of a unimolecular iminocoumarin-benzothiazole derivative to biomolecular segments as reported by Kim et al.[4] Latent fluorescence signals of iminocoumarin benzothiazole recommenced in living cells because cellular alkaline phosphatase catalysis led to release of phosphate groups. **Figure S4**. Illustration of a topo I binding site-containing plasmid DNA, pHOT1, and topo I-catalyzed conversion from its supercoiled form to its relaxed form. **Figure S5**. Illustration of molecular structures of fluorophore Cy3 (a), quencher BHQ-2 (b) and phosphorothioate (c) used in the current studies. **Figure S6**. Illustration of nucleotide sequences and modifications of Oligonucleotide 1 and Oligonucleotide 2 as well as synthesis of Probe 1 from Oligonucleotide 1 and Oligonucleotide 2 catalyzed by T4 DNA ligase. **Figure S7**. Characterization of the structural difference between Probe 1 and Probe 2 by exonuclease digestion. Lanes 1-4, mixtures containing 3 pmol of DNA-based biosensors (Probe 1 for Lanes 1 and 2, Probe 2 for Lanes 3 and 4), 67 mM glycine-KOH (pH 9.4), 2.5 mM MgCl2 and 50 μg/ml BSA were incubated in the absence (Lanes 1 and 3) or presence (Lanes 2 and 4) of 1 U of lambda exonuclease at 37 °C for 15 min. Polyacrylamide gel electrophoresis was carried out using a 15 % non-denaturing gel in Tris-boric-EDTA buffer at 10 V/cm for 2 h, followed by ethidium bromide staining. **Figure S8**. Verification of the dumbbell-shaped DNA structure by restriction enzyme digestion. Lanes 1-2, mixtures containing 3 pmol of Probe 1 and 1X rCutSmart Buffer were incubated in the absence (Lanes 1) or presence (Lanes 2) of 1 U of MboI at 37 °C for 15 min. Polyacrylamide gel electrophoresis was carried out using a 15 % non-denaturing gel in Tris-boric-EDTA buffer at 10 V/cm for 2 h, followed by ethidium bromide staining. **Figure S9**. (a) Illustration of structural differences of Probe 1 from Probes 4 to 7. (b) Correlation of relative fluorescence intensities of Probes 4 to 7 in the presence of human topo I. The value of (F - F0) / F0 indicates the change in fluorescence intensity relative to baseline, where F0 and F represent intensities of the fluorescence at the wavelength of 565 nm before and after enzymatic reactions, respectively. **Figure S10**. (a) Illustration of topo I-catalyzed formation of Fragment 1 and Fragment 2 from Probe 1. (b) Electrophoretic analysis of reaction mixtures of Probe 1 in the presence of topo I. A solution containing 50 nM of Probe 1, 10 mM Tris-HCl (pH 7.5), 1 mM EDTA, 100 mM NaCl, 0.1% BSA, 0.1 mM spermidine, and 5% glycerol was kept at 37 °C for 1 hour in the presence of different amounts of human topo I. The resultant solutions were then mixed with DNA loading buffers and loaded onto a 15% native polyacrylamide gel in 1× TBE buffer (Tris-borate-EDTA). O’RangeRuler 5 bp (Thermo Fisher Scientific) as a double-stranded DNA molecular weight marker was loaded onto the polyacrylamide gels in parallel (Lane 1). The polyacrylamide gel was further run at 15 V/cm for 3 hours followed by staining with 0.5 μg/ml ethidium bromide and visualization under UV illumination using a G:BOX iChemi gel documentation apparatus (Syngene). The concentrations of topo I used in these studies were 0 nM (Lane 2), 0.5 nM (Lane 3), 1 nM (Lane 4), 2 nM (Lane 5) and 5 nM (Lane 6). **Figure S11**. (a) Structural comparison of Probe 1 with Probe 2 and Probe 3. (b) Fluorescence spectra of topo I-catalyzed reaction mixtures of Probe 1. (c) Anticipated catalytic pathways of topo I-catalyzed reaction on Probe 1. (d) Fluorescence spectra of topo I-catalyzed reaction mixtures of Probe 2. (e) Anticipated catalytic pathways of topo I-catalyzed reaction on Probe 2. In this process, topo I is covalently trapped stoichiometrically upon completion of Step 3 so that this enzyme is unable to proceed with its new rounds of catalysis. (f) Fluorescence spectra of topo I-catalyzed reaction mixtures of Probe 3. (g) Anticipated catalytic pathways of topo I-catalyzed reaction on Probe 3. In this process, topo I is covalently trapped stoichiometrically upon completion of Step 4 so that this enzyme is unable to proceed with its new rounds of catalysis. **Figure S12**. The relative level of topo I in lysates of the topo I-upregulated and the ordinary HEK293T cells determined via ELISA assay. Data are expressed as the mean ± standard deviation (*P < 0.005; n = 3). **Figure S13**. Examination of the cytotoxicity of the DNA-based biosensors and the cationic liposome nanocarriers. Cell viabilities were determined by MTT assay (shown as the mean ± SD, n = 3) and expressed as percentages of those of control (without probes and liposomes). Amounts of liposomes in each column were 0.08, 0.16, 0.4, 0.8, 1.6 ul, respectively. HT-29 cells were incubated with Probe 1 and/or Lipofectamine LTX at 37 °C for 36 h before MTT assay. **Figure S14**. The relative level of topo I in HT-29 and topo I gene-knockdown HT-29 cell lines determined via ELISA assay. Data are expressed as the mean ± standard deviation (*P < 0.005; n = 3). **Table S1**. Nucleotide sequences of oligonucleotides used in this study and their modifications.

## Data Availability

All data and materials are available within the manuscript and in additional files.
