# Supplementary material for: Tricking enzymes in living cells: a mechanism-based strategy for design of DNA topoisomerase biosensors
Source: J Nanobiotechnology. 2021 Dec 7;19:407. doi: 10.1186/s12951-021-01155-1 (PMC8650243; doi:10.1186/s12951-021-01155-1)
Supplement: Supplementary file 1 — Additional file 1: Figure S1. Comparison between enzymatic reactions catalyzed by most cellular enzymes and DNA topoisomerases. (a) Schematic illustration of an enzyme-catalyzed reaction starting with fluorophore-quencher-containing substrates. In this process, fluorescence signals of a quenched fluorophore recommence owing to generation of bimolecular entities. (b) Schematic illustration of a human topoisomerase-catalyzed reaction. In the catalytic reaction, DNA topoisomerases causes conformational differences between its substrate (supercoiled form DNA) and product (relaxed form DNA) while molecular configurations of them remain unchanged. Figure S2. Caspase-catalyzed conversion of a unimolecular peptide derivative to biomolecular segments as reported by Kool group.[3] Because cellular caspase-catalyzed covalent bond breakage resulted in separation of tetrapyrene from dabcyl, quenched fluorescence signals of tetrapyrene recommenced in living cells. Figure S3. Alkaline phosphatase-catalyzed conversion of a unimolecular iminocoumarin-benzothiazole derivative to biomolecular segments as reported by Kim et al.[4] Latent fluorescence signals of iminocoumarin benzothiazole recommenced in living cells because cellular alkaline phosphatase catalysis led to release of phosphate groups. Figure S4. Illustration of a topo I binding site-containing plasmid DNA, pHOT1, and topo I-catalyzed conversion from its supercoiled form to its relaxed form. Figure S5. Illustration of molecular structures of fluorophore Cy3 (a), quencher BHQ-2 (b) and phosphorothioate (c) used in the current studies. Figure S6. Illustration of nucleotide sequences and modifications of Oligonucleotide 1 and Oligonucleotide 2 as well as synthesis of Probe 1 from Oligonucleotide 1 and Oligonucleotide 2 catalyzed by T4 DNA ligase. Figure S7. Characterization of the structural difference between Probe 1 and Probe 2 by exonuclease digestion. Lanes 1-4, mixtures containing 3 pmol of DNA-based biosensors (Probe 1 [file 12951_2021_1155_MOESM1_ESM.pdf]

Supporting Information

Tricking Enzymes in Living Cells: A Mechanism-Based Strategy for Design of DNA Topoisomerase Biosensors

Sai Ba[1,2†], Guangpeng Gao[1†], Tianhu Li[1] and Hao Zhang[1,2*]*

**Correspondence: tianhuli@nwpu.edu.cn; zhanghaolab@nwpu.edu.cn*

[1] School of Chemistry and Chemical Engineering Northwestern Polytechnical University, Xi'an 710072, China

[2] Division of Chemistry and Biological Chemistry, School of Physical and Mathematical Sciences, Nanyang Technological University, Singapore 637371

Materials

DNA oligonucleotides that contained modifications of fluorophores, quenchers, 5' phosphorylation and phosphorothioate modifications were purchased from Sangon Biotech (Shanghai, China). Detailed structures and nucleotide sequences of oligonucleotides used in the current studies are listed in Figure S5 and Table S1. Upon arrival, all the oligonucleotides were prepared into 100 μM stock solutions through using deionized water as solvent and stored at -20 °C prior to use. Human topoisomerase I (topo I), human topoisomerase IIα, *E. coli* topoisomerase IV, *E. coli* DNA gyrase were purchased from TopoGEN Inc (Buena Vista, CO) while *E. coli* topoisomerase I and T4 DNA ligase were the commercial products of New England Biolabs (Singapore). Non-tumorigenic human colon cells (CCD-18Co cell line) and human colon cancer cells (HT-29 cell line) were obtained from American Type Culture Collection (Manassas, VA). Media, supplements and reagents (McCoy's 5A (Modified) Medium, Eagle's Minimum Essential Medium (EMEM), fetal bovine serum, trypsin-EDTA (0.25%), phosphate-buffered saline (PBS), NucBlue Live ReadyProbes reagent, Lipofectamine LTX with PLUS Reagent, Lipofectamine RNAiMAX and Vybrant MTT cell proliferation assay kit) used for cell culture and cytotoxicity assays were purchased from Thermo Fisher Scientific (Waltham, MA). Cell culture and imaging dishes (35-mm μ-Dish with Ibidi polymer coverslip bottom) for confocal microscopic examinations were provided by Ibidi GmbH (Martinsried, Germany). Chemically synthesized small interfering RNA (ON-TARGETplus Human TOP1 siRNA) for preparation of FEN1 gene-silenced cells was purchased from Dharmacon (Lafayette, CO). Topo I overexpression cell lysates and empty vector transfected control cell lysates were obtained from Novus Biologicals (Centennial, CO). ELISA kit for human topo I was purchased from Cloud-Clone Corp (Katy, TX). Small-molecule inhibitors of topo I, topotecan and irinotecan, and other reagents used in this study (*e.g.* chemicals, buffers, electrophoresis reagents) were purchased from Sigma-Aldrich Pte Ltd (Singapore).

Supplementary Figures

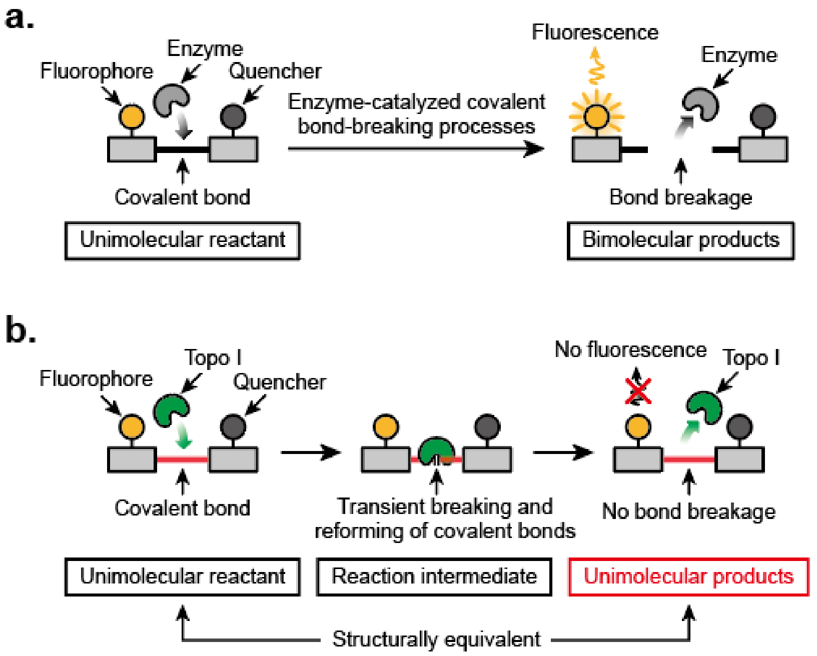

Figure S1. Comparison between enzymatic reactions catalyzed by most cellular enzymes and DNA topoisomerases. (a) Schematic illustration of an enzyme-catalyzed reaction starting with fluorophore-quencher-containing substrates. In this process, fluorescence signals of a quenched fluorophore recommence owing to generation of bimolecular entities. (b) Schematic illustration of a human topoisomerase-catalyzed reaction. In the catalytic reaction, DNA topoisomerases causes conformational differences between its substrate (supercoiled form DNA) and product (relaxed form DNA) while molecular configurations of them remain unchanged.

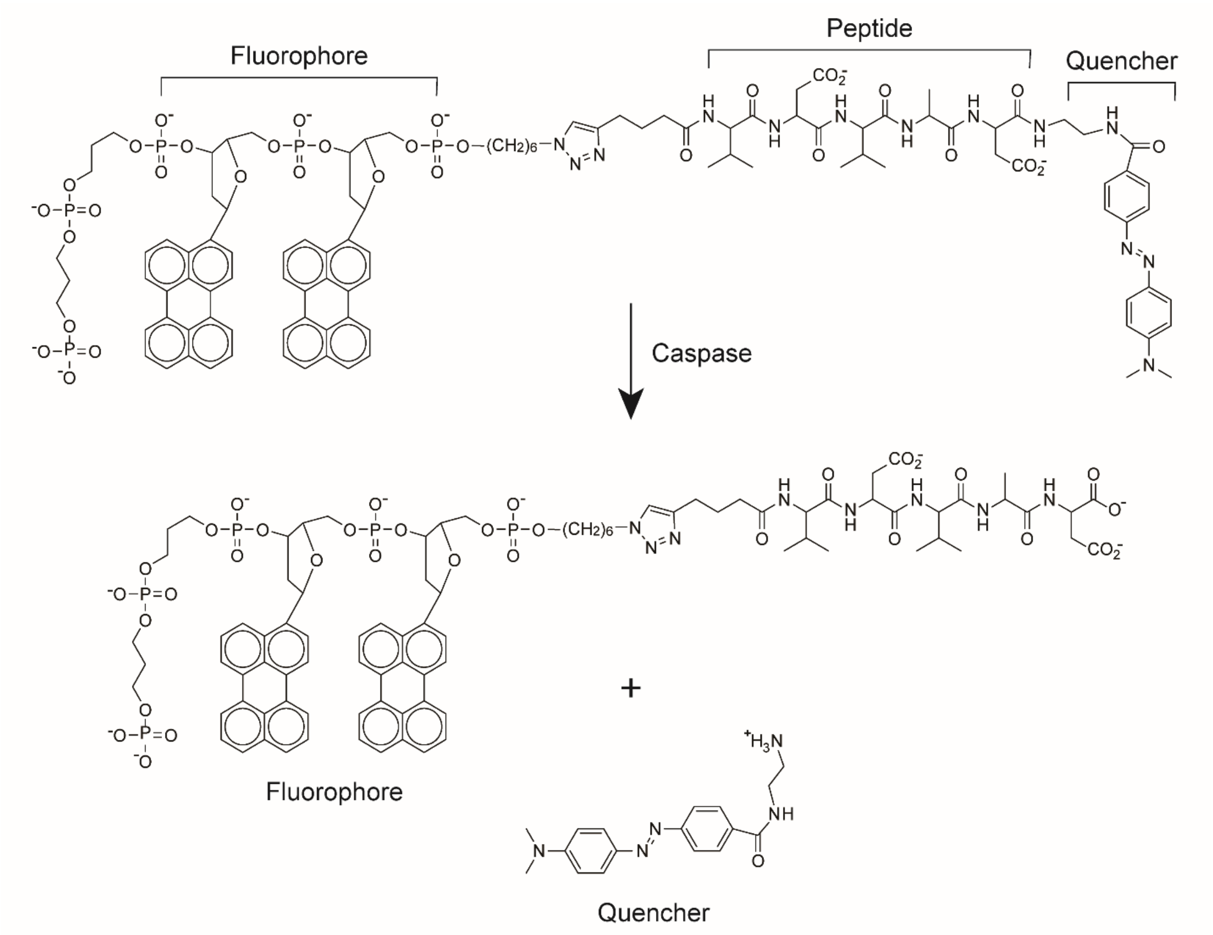

Figure S2. Caspase-catalyzed conversion of a unimolecular peptide derivative to biomolecular segments as reported by Kool group.[3] Because cellular caspase-catalyzed covalent bond breakage resulted in separation of tetrapyrene from dabcyl, quenched fluorescence signals of tetrapyrene recommenced in living cells.

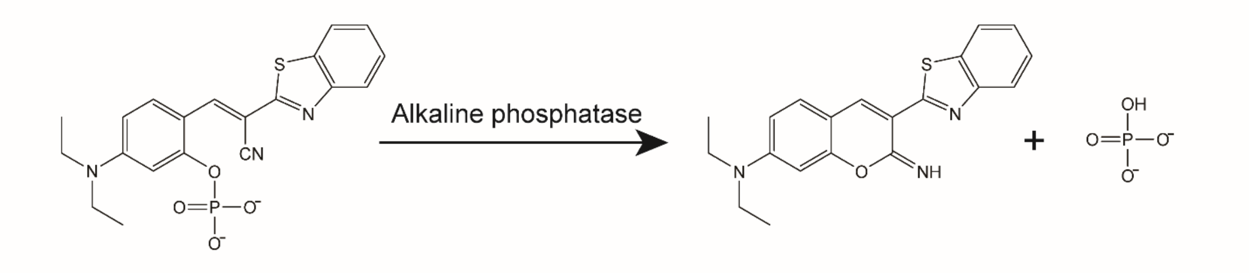

Figure S3. Alkaline phosphatase-catalyzed conversion of a unimolecular iminocoumarin-benzothiazole derivative to biomolecular segments as reported by Kim *et al.*[4] Latent fluorescence signals of iminocoumarin benzothiazole recommenced in living cells because cellular alkaline phosphatase catalysis led to release of phosphate groups.

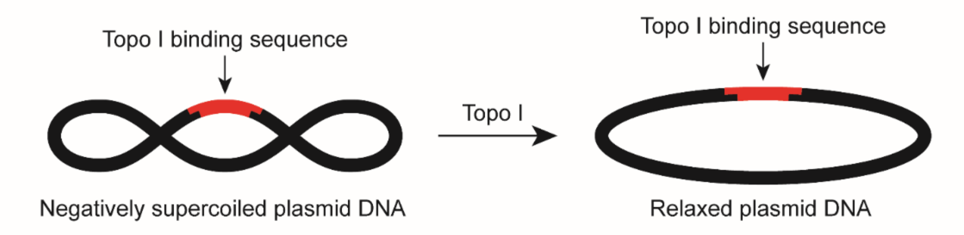

Figure S4. Illustration of a topo I binding site-containing plasmid DNA, pHOT1, and topo I-catalyzed conversion from its supercoiled form to its relaxed form.

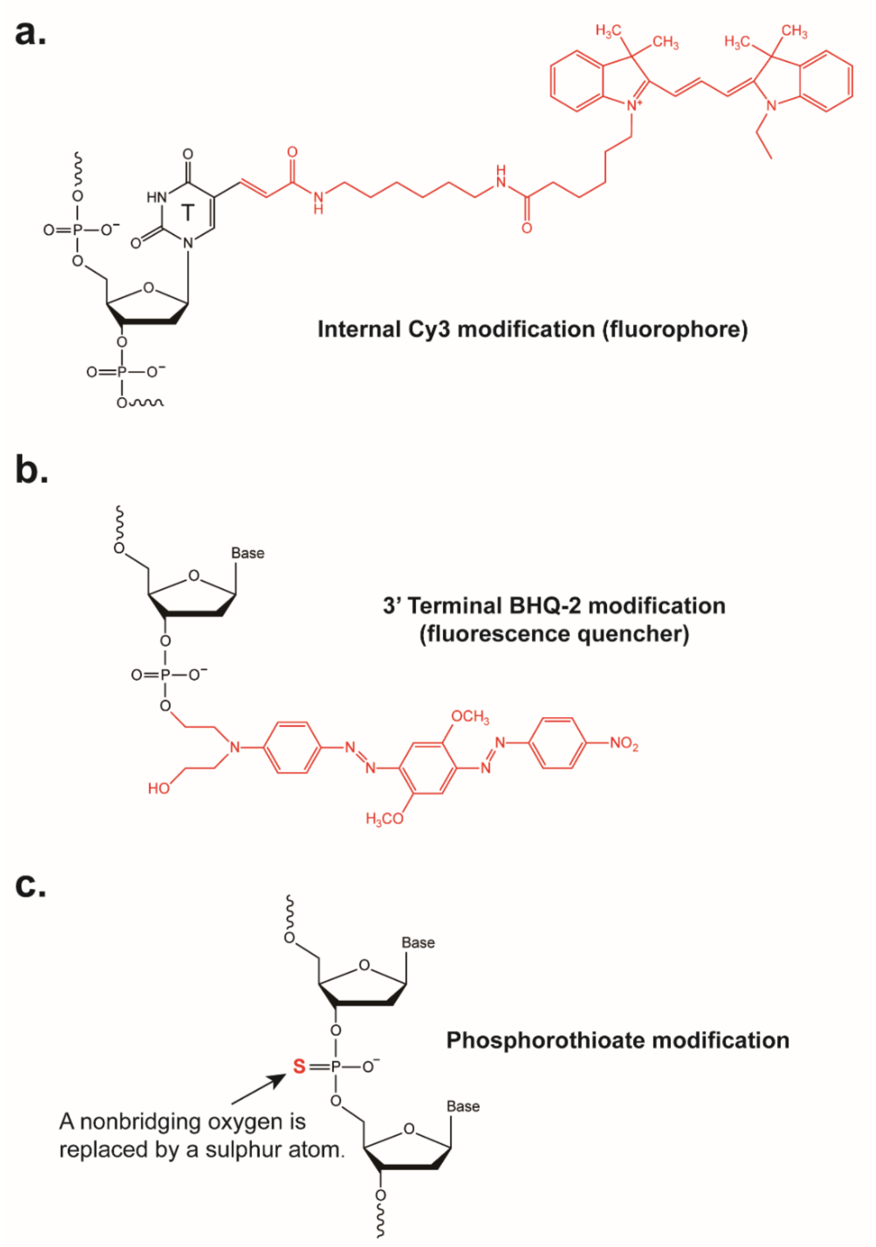

Figure S5. Illustration of molecular structures of fluorophore Cy3 (a), quencher BHQ-2 (b) and phosphorothioate (c) used in the current studies.

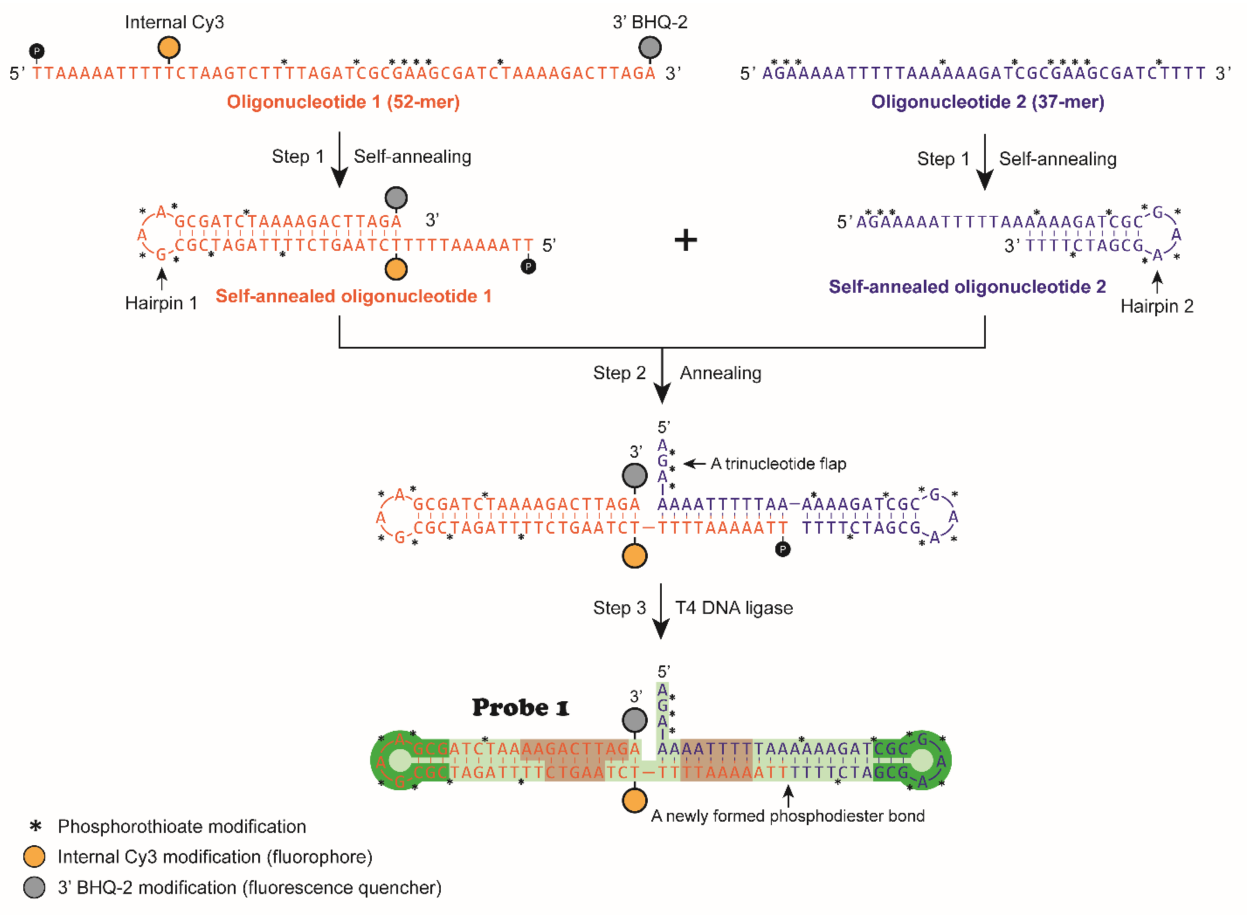

Figure S6. Illustration of nucleotide sequences and modifications of Oligonucleotide 1 and Oligonucleotide 2 as well as synthesis of Probe 1 from Oligonucleotide 1 and Oligonucleotide 2 catalyzed by T4 DNA ligase.

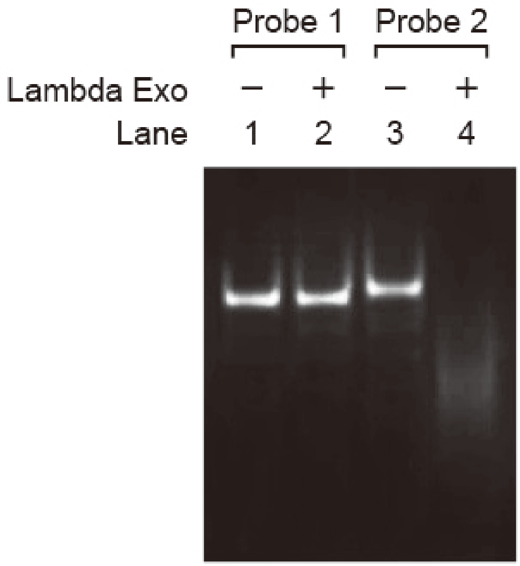

Figure S7. Characterization of the structural difference between Probe 1 and Probe 2 by exonuclease digestion. Lanes 1-4, mixtures containing 3 pmol of DNA-based biosensors (Probe 1 for Lanes 1 and 2, Probe 2 for Lanes 3 and 4), 67 mM glycine-KOH (pH 9.4), 2.5 mM $MgCl_2$ and 50 μg/ml BSA were incubated in the absence (Lanes 1 and 3) or presence (Lanes 2 and 4) of 1 U of lambda exonuclease at 37 °C for 15 min. Polyacrylamide gel electrophoresis was carried out using a 15 % non-denaturing gel in Tris-boric-EDTA buffer at 10 V/cm for 2 h, followed by ethidium bromide staining.

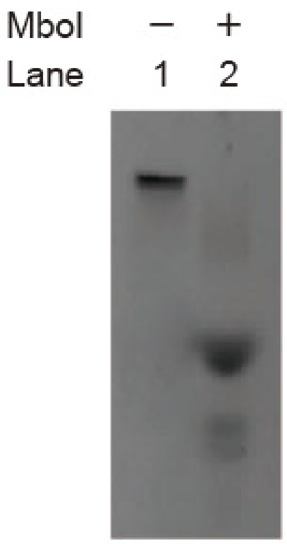

Figure S8. Verification of the dumbbell-shaped DNA structure by restriction enzyme digestion. Lanes 1-2, mixtures containing 3 pmol of Probe 1 and 1X rCutSmart Buffer were incubated in the absence (Lanes 1) or presence (Lanes 2) of 1 U of MboI at 37 °C for 15 min. Polyacrylamide gel electrophoresis was carried out using a 15 % non-denaturing gel in Tris-boric-EDTA buffer at 10 V/cm for 2 h, followed by ethidium bromide staining.

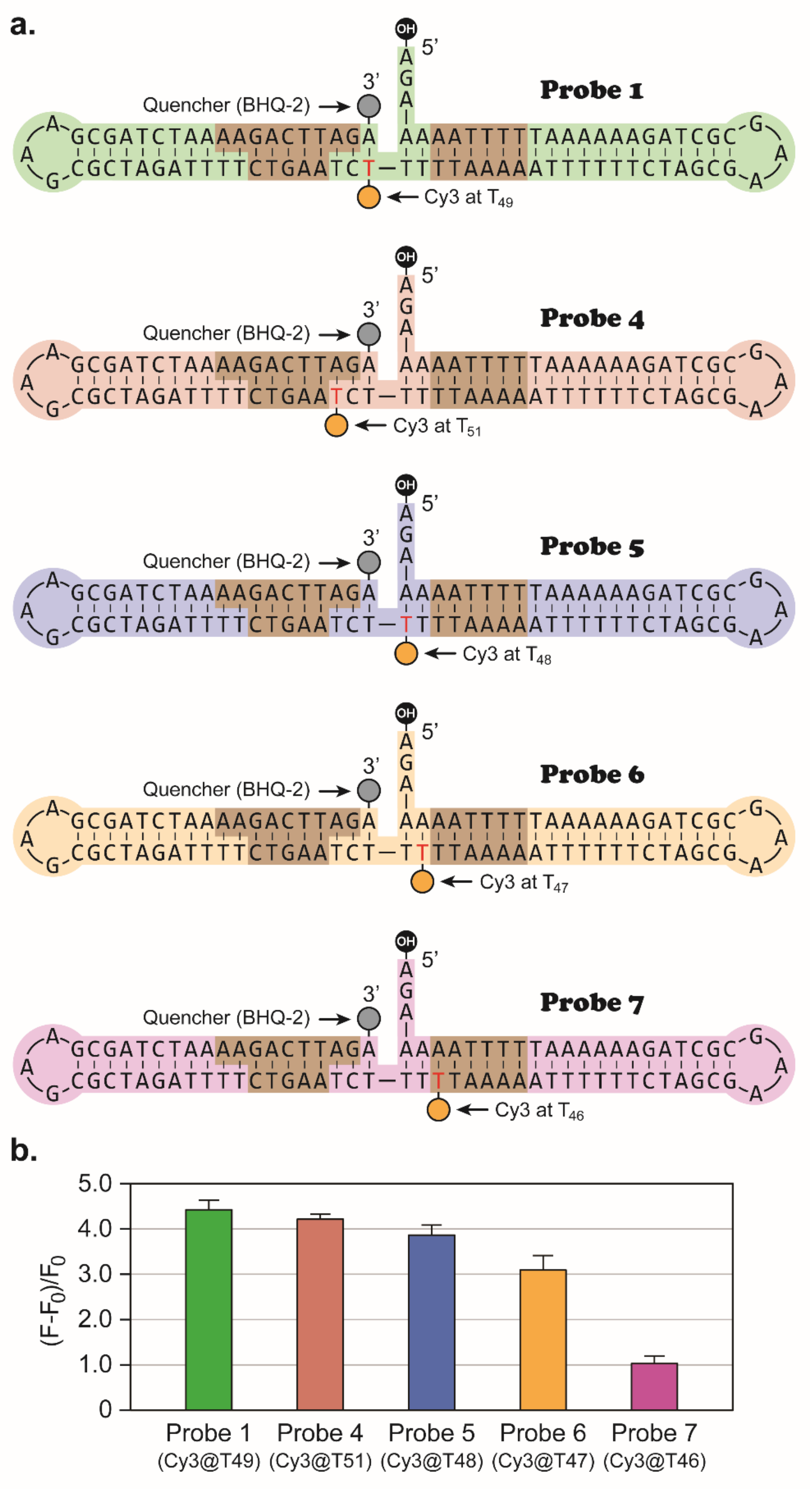

Figure S9. (a) Illustration of structural differences of Probe 1 from Probes 4 to 7. (b) Correlation of relative fluorescence intensities of Probes 4 to 7 in the presence of human topo I. The value of (F - F0) / F0 indicates the change in fluorescence intensity relative to baseline, where F_0 and F represent intensities of the fluorescence at the wavelength of 565 nm before and after enzymatic reactions, respectively.

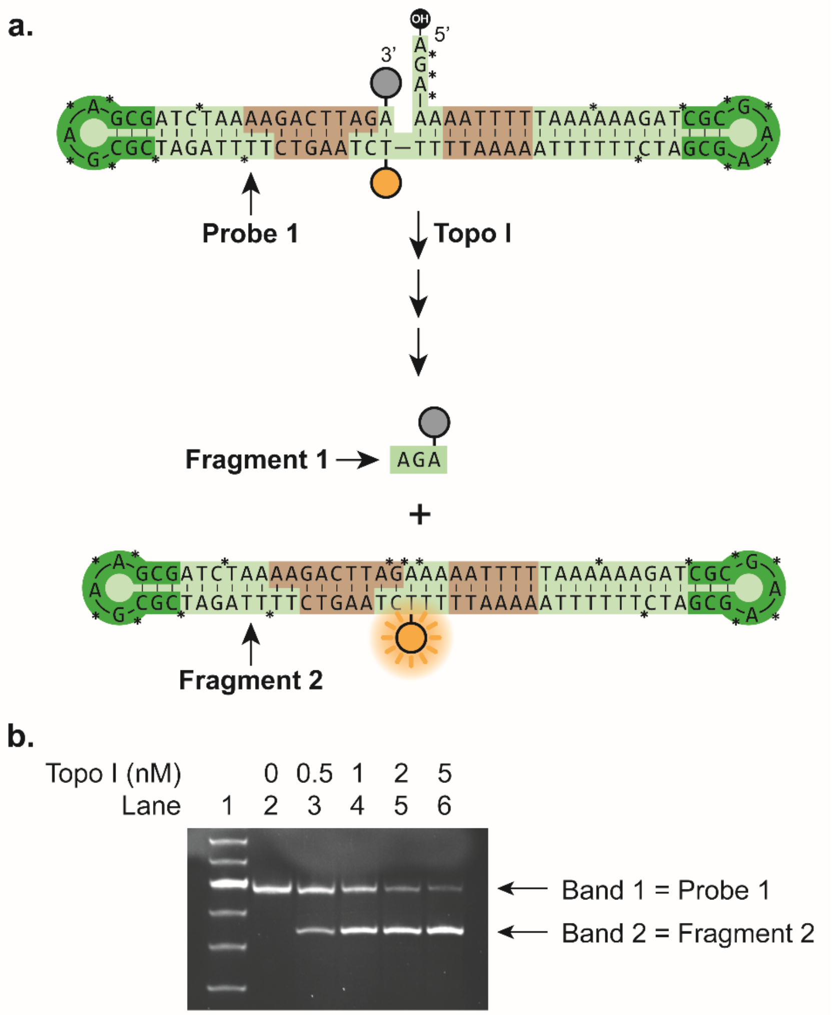

Figure S10. (a) Illustration of topo I-catalyzed formation of Fragment 1 and Fragment 2 from Probe 1. (b) Electrophoretic analysis of reaction mixtures of Probe 1 in the presence of topo I. A solution containing 50 nM of Probe 1, 10 mM Tris-HCl (pH 7.5), 1 mM EDTA, 100 mM NaCl, 0.1% BSA, 0.1 mM spermidine, and 5% glycerol was kept at 37 °C for 1 hour in the presence of different amounts of human topo I. The resultant solutions were then mixed with DNA loading buffers and loaded onto a 15% native polyacrylamide gel in 1× TBE buffer (Tris-borate-EDTA). O'RangeRuler 5 bp (Thermo Fisher Scientific) as a double-stranded DNA molecular weight marker was loaded onto the polyacrylamide gels in parallel (Lane 1). The polyacrylamide gel was further run at 15 V/cm for 3 hours followed by staining with 0.5 μg/ml ethidium bromide and visualization under UV illumination using a G:BOX iChemi gel documentation apparatus (Syngene). The concentrations of topo I used in these studies were 0 nM (Lane 2), 0.5 nM (Lane 3), 1 nM (Lane 4), 2 nM (Lane 5) and 5 nM (Lane 6).

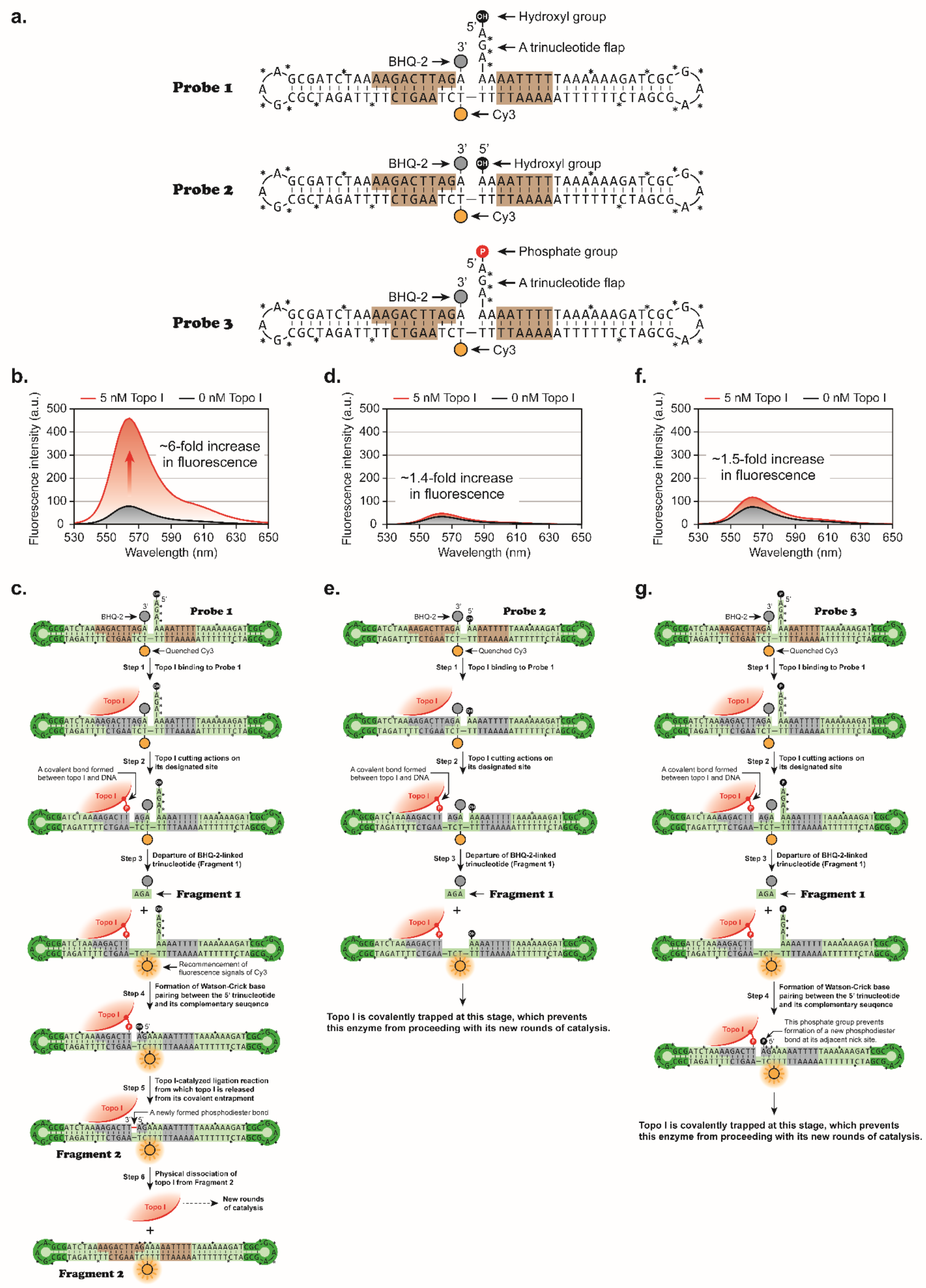

a.
Probe 1
Probe 2
Probe 3
Hydroxyl group
A trinucleotide flap
Phosphate group
BHQ-2
Cy3
b.
d.
f.
5 nM Topo I
0 nM Topo I
Fluorescence intensity (a.u.)
Wavelength (nm)
~6-fold increase in fluorescence
~1.4-fold increase in fluorescence
~1.5-fold increase in fluorescence
c.
e.
g.
Quenched Cy3
Step 1 Topo I binding to Probe 1
Step 2 Topo I cutting actions on its designated site
A covalent bond formed between topo I and DNA
Step 3 Departure of BHQ-2-linked trinucleotide (Fragment 1)
Fragment 1
Recommencement of fluorescence signals of Cy3
Step 4 Formation of Watson-Crick base pairing between the 5' trinucleotide and its complementary seuqence
Step 5 Topo I-catalyzed ligation reaction from which topo I is released from its covalent entrapment
A newly formed phosphodiester bond
Fragment 2
Step 6 Physical dissociation of topo I from Fragment 2
New rounds of catalysis
Topo I is covalently trapped at this stage, which prevents this enzyme from proceeding with its new rounds of catalysis.
Step 4 Formation of Watson-Crick base pairing between the 5' trinucleotide and its complementary seuqence
This phosphate group prevents formation of a new phosphodiester bond at its adjacent nick site.
Topo I is covalently trapped at this stage, which prevents this enzyme from proceeding with its new rounds of catalysis.

Figure S11. (a) Structural comparison of Probe 1 with Probe 2 and Probe 3. (b) Fluorescence spectra of topo I-catalyzed reaction mixtures of Probe 1. (c) Anticipated catalytic pathways of topo I-catalyzed reaction on Probe 1. (d) Fluorescence spectra of topo I-catalyzed reaction mixtures of Probe 2. (e) Anticipated catalytic pathways of topo I-catalyzed reaction on Probe 2. In this process, topo I is covalently trapped stoichiometrically upon completion of Step 3 so that this enzyme is unable to proceed with its new rounds of catalysis. (f) Fluorescence spectra of topo I-catalyzed reaction mixtures of Probe 3. (g) Anticipated catalytic pathways of topo I-catalyzed reaction on Probe 3. In this process, topo I is covalently trapped stoichiometrically upon completion of Step 4 so that this enzyme is unable to proceed with its new rounds of catalysis.

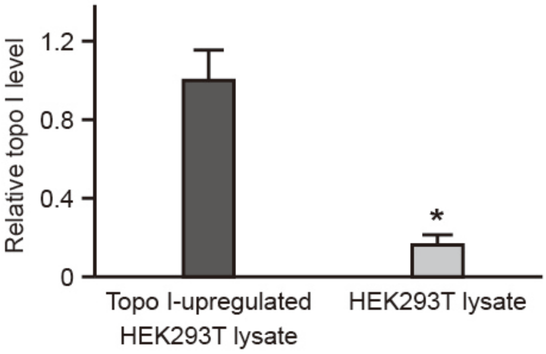

Figure S12. The relative level of topo I in lysates of the topo I-upregulated and the ordinary HEK293T cells determined via ELISA assay. Data are expressed as the mean ± standard deviation (*P < 0.005; n = 3).

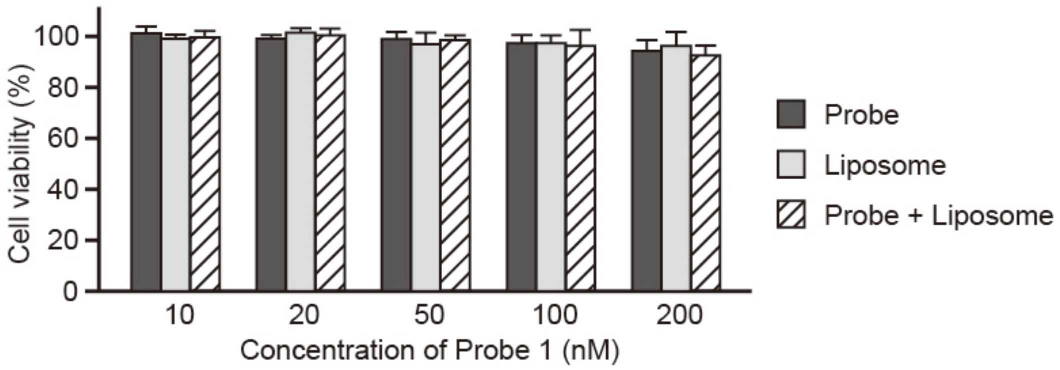

Figure S13. Examination of the cytotoxicity of the DNA-based biosensors and the cationic liposome nanocarriers. Cell viabilities were determined by MTT assay (shown as the mean ± SD, n = 3) and expressed as percentages of those of control (without probes and liposomes). Amounts of liposomes in each column were 0.08, 0.16, 0.4, 0.8, 1.6 ul, respectively. HT-29 cells were incubated with Probe 1 and/or Lipofectamine LTX at 37 °C for 36 h before MTT assay.

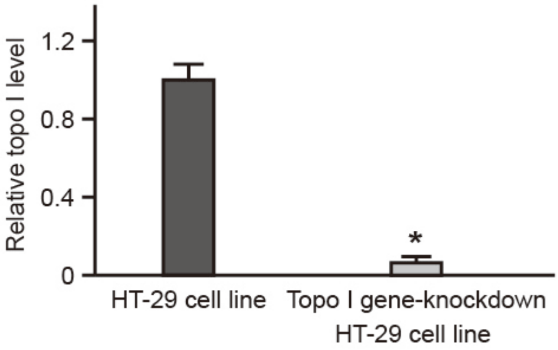

Figure S14. The relative level of topo I in HT-29 and topo I gene-knockdown HT-29 cell lines determined via ELISA assay. Data are expressed as the mean ± standard deviation (*P < 0.005; n = 3).

Supplementary Tables

Table S1. Nucleotide sequences of oligonucleotides used in this study and their modifications.

Name	Nucleotide sequence (5' to 3')	Structural modification*
Oligonucleotide 1	[Phos]TTAAAAATTTT**<u>T</u>**CTAAGTCTT^<br>TTAGAT^CGC^G^A^A^GCGATC^TAA<br>AAGACTTAGA[BHQ-2]	5' Phosphorylation [Phos];<br>3' BHQ-2 [BHQ-2];<br>Cy3-modified thymine [**<u>T</u>**];<br>Phosphorothioate [^]
Oligonucleotide 2	A^G^A^AAAATTTTTAAA^AAAGAT^<br>CGC^G^A^A^GCGATC^TTTT	Phosphorothioate [^]
Oligonucleotide 3	AAAATTTTTAAA^AAAGAT^CGC^G^<br>A^A^GCGATC^TTTT	Phosphorothioate [^]
Oligonucleotide 4	[Phos]A^G^A^AAAATTTTTAAA^AAA<br>GAT^CGC^G^A^A^GCGATC^TTTT	5' Phosphorylation [Phos];<br>Phosphorothioate [^]
Oligonucleotide 5	[Phos]TTAAAAATTTTTC**<u>T</u>**AAGTCTT^<br>TTAGAT^CGC^G^A^A^GCGATC^TAA<br>AAGACTTAGA[BHQ-2]	5' Phosphorylation [Phos];<br>3' BHQ-2 [BHQ-2];<br>Cy3-modified thymine [**<u>T</u>**];<br>Phosphorothioate [^]
Oligonucleotide 6	[Phos]TTAAAAATTT**<u>T</u>**TCTAAGTCTT^<br>TTAGAT^CGC^G^A^A^GCGATC^TAA<br>AAGACTTAGA[BHQ-2]	5' Phosphorylation [Phos];<br>3' BHQ-2 [BHQ-2];<br>Cy3-modified thymine [**<u>T</u>**];<br>Phosphorothioate [^]
Oligonucleotide 7	[Phos]TTAAAAATT**<u>T</u>**TTCTAAGTCTT^<br>TTAGAT^CGC^G^A^A^GCGATC^TAA<br>AAGACTTAGA[BHQ-2]	5' Phosphorylation [Phos];<br>3' BHQ-2 [BHQ-2];<br>Cy3-modified thymine [**<u>T</u>**];<br>Phosphorothioate [^]
Oligonucleotide 8	[Phos]TTAAAAAT**<u>T</u>**TTTCTAAGTCTT^<br>TTAGAT^CGC^G^A^A^GCGATC^TAA<br>AAGACTTAGA[BHQ-2]	5' Phosphorylation [Phos];<br>3' BHQ-2 [BHQ-2];<br>Cy3-modified thymine [**<u>T</u>**];<br>Phosphorothioate [^]

* Detailed structures of chemical modifications are shown in Figure S5.

References

1. N. Dai, J. Guo, Y. N. Teo and E. T. Kool, *Angewandte Chemie International Edition*, 2011, **50**, 5105-5109.
2. T.-I. Kim, H. Kim, Y. Choi and Y. Kim, *Chemical Communications*, 2011, **47**, 9825-9827.
3. L. Guo, X. Liu, Y. Jiang, K. Nishikawa and W. Plunkett, *Molecular pharmacology*, 2011, **80**, 321-327.
4. H. J. Park, H.-J. Lee, E.-J. Lee, H. J. Hwang, S.-H. Shin, M.-E. Suh, C. Kim, H. J. Kim, E.-K. Seo and S. K. Lee, *Bioscience, Biotechnology, and Biochemistry*, 2003, **67**, 1944-1949.
